# Precision medicine with car cells in acute myeloid leukemia: where are we?

**DOI:** 10.3389/fimmu.2025.1653350

**Published:** 2025-11-06

**Authors:** Larissa C. Zanetti, Victoria Tomaz, Ingrid Ferreira de Souza, Paulo V. Campregher, Nelson Hamerschlak, Lucila N. Kerbauy

**Affiliations:** 1 Hospital Israelita Albert Einstein, São Paulo, Brazil; 2 Genesis Genomics, São Paulo, Brazil

**Keywords:** CAR-T, AML, immunotherapy, precision medicine, genetic mutations

## Abstract

The integration of chimeric antigen receptor (CAR) therapies with precision medicine holds potential to impact the treatment landscape for acute myeloid leukemia (AML). Genetic mutations play a role in the efficacy of CAR-T and CAR-NK cells, influencing their crucial role in determining the effectiveness of these cells, as well as their proliferation, persistence, resistance, and safety. This review examines how mutations in FLT3, DNMT3A, NPM1, TP53, TET2, gene fusions involving RUNX1 and KMT2A and other key genes modulate CAR-based immunotherapies, highlighting both vulnerabilities and resistance mechanisms. Recent findings demonstrate that mutations in genes such as DNMT3A and NPM1 enhance antigen expression, thereby improving CAR targeting. In contrast, mutations in TP53 drive immune escape and resistance to therapy. Understanding these mutation-specific effects is essential for tailoring CAR therapies to individual patients, optimizing efficacy while minimizing toxicity. By leveraging genomic profiling and personalized engineering approaches, CAR therapies can be refined to overcome resistance and enhance precision in AML treatment. Future research should focus on integrating multiomic data to develop mutation-adapted CAR strategies, ensuring that patients receive the most effective and personalized immunotherapy.

## Introduction

Precision medicine in onco-hematology has been revolutionized by the development of chimeric antigen receptor (CAR) cell therapies, particularly with the use of CAR-modified T cells (CAR-T) and CAR-modified natural killer cells (CAR-NK). These approaches have introduced a highly targeted strategy for treating hematologic malignancies, offering new hope for patients with relapsed or refractory disease, especially in acute lymphoblastic leukemia (1), non-Hodgkin lymphoma (2), and multiple myeloma (3). Unlike conventional treatments, which often lack specificity and result in widespread cytotoxicity, CAR therapies provide a personalized approach by engineering immune cells to recognize and eliminate malignant cells based on specific surface antigens (4). The success of CAR-T cell therapy has been exemplified by FDA-approved treatments targeting CD19 and BCMA in B-cell malignancies (5–11).

CAR-T cells are generated by modifying a patient’s T lymphocytes to express a synthetic receptor specific to an antigen present on cancer cells (12–14). This modification enhances the ability of T cells to recognize and kill tumor cells with high specificity (15). Clinical trials have demonstrated impressive response rates, including complete remissions in cases previously resistant to conventional therapies (16). Although immune-effector cell-associated neurotoxicity syndrome (ICANS) and cytokine release syndrome (CRS) are now better managed and less concerning to clinicians, significant challenges persist (17–19), These include concerns about late adverse effects such as secondary neoplasias, particularly secondary myeloid neoplasms, which have been reported in some cases but have not been definitively proven to be caused by CAR-T cell therapy (20–24). Additional major challenges include the logistical timeframe from lymphocyte apheresis to CAR-T cell delivery, patient lymphodepletion protocols, and the standardization of apheresis strategies for manufacturing, all of which significantly affect treatment success and patient management (25–29), Additionally, tumor escape mechanisms such as antigen loss or downregulation continue to complicate therapeutic efficacy (19).

To address the limitations of CAR-T therapy, CAR-NK cell therapy has emerged as a promising alternative. Natural killer (NK) cells are innate immune effectors capable of directly killing tumor cells without prior antigen sensitization (30–32). CAR-NK cells can be derived from multiple sources, including cord blood, peripheral blood, and established NK cell lines such as NK-92 (33). Compared to CAR-T cells, CAR-NK cells show a more favorable safety profile, with a significantly reduced risk of CRS and ICANS due to their more regulated cytokine secretion (34, 35). Importantly, CAR-NK cells hold manufacturing advantages as an “off-the-shelf” product: they can be produced from universal donors without the need for individualized patient-specific engineering, allowing scalable manufacturing, rapid availability, and reduced costs (33–37).

Recent advances have also addressed the historical limitation of NK cell persistence *in vivo*. Genetic engineering approaches including co-expression of cytokines like IL-15 have extended CAR-NK survival and proliferation, enabling detectable persistence in patients for up to a year in some clinical contexts (34). Preclinical and early clinical data demonstrate promising anti-tumor efficacy of CAR-NK cells targeting BCMA, CD19, and other tumor-associated antigens, with a lower incidence of graft-versus-host disease (GVHD) relative to allogeneic CAR-T therapies (34, 37–39). Nonetheless, challenges remain, including optimizing *in vivo* persistence, improving trafficking to solid tumors, and remaining vigilant for any off-target or long-term safety effects (39, 40).

Overall, CAR-NK therapy represents a compelling and complementary cellular immunotherapy platform that combines enhanced safety, off-the-shelf manufacturing scalability, and improving persistence, positioning it as a pivotal option in the future immuno-oncology arsenal. However, since CAR-NK trials are still recent, the literature on the effect of AML mutations on CAR-NK cell therapy remains scarce.

Among hematologic malignancies, acute myeloid leukemia (AML) remains a particularly challenging disease due to its high relapse rates and poor long-term survival outcomes. AML is characterized by significant genetic and antigenic heterogeneity, making the identification of universal CAR targets challenging (41–43). Furthermore, many potential AML-associated antigens, such as CD33 and CD123, are also expressed on healthy hematopoietic stem and progenitor cells, increasing the risk of on-target, off-tumor toxicity (44). Despite these challenges, ongoing research into CAR therapies for AML has identified promising targets, including CD33 (45), CD123 (46), and CLL-1 (47), among others (48–62). Additionally, mutations such as FLT3-ITD, NPM1, and DNMT3A not only contribute to AML pathogenesis but may also influence responses to CAR-based therapies, emphasizing the need for molecularly guided treatment strategies.

This review will examine the latest advancements in CAR-T and CAR-NK therapies, with a particular focus on their application in precision medicine for AML. We will discuss the influence of molecular subtypes on therapeutic efficacy, explore the key challenges in CAR therapy for AML, and highlight future directions for integrating CAR-based treatments into personalized treatment frameworks for hematologic malignancies.

## The role of molecular alterations in leukemogenesis

Hematopoietic stem cells (HSCs), due to their long lifespan and self-renewal capacity, are particularly prone to accumulating mutations over time, which can drive clonal evolution toward hematologic malignancies. Although most spontaneous mutations have minimal clinical impact or are efficiently eliminated by immune surveillance, specific genetic alterations affecting key regulators of hematopoiesis can disrupt critical signaling and transcriptional networks. These changes confer a selective growth advantage to mutated clones, promoting their expansion and ultimately contributing to leukemogenesis (63).

**Figure 1:**
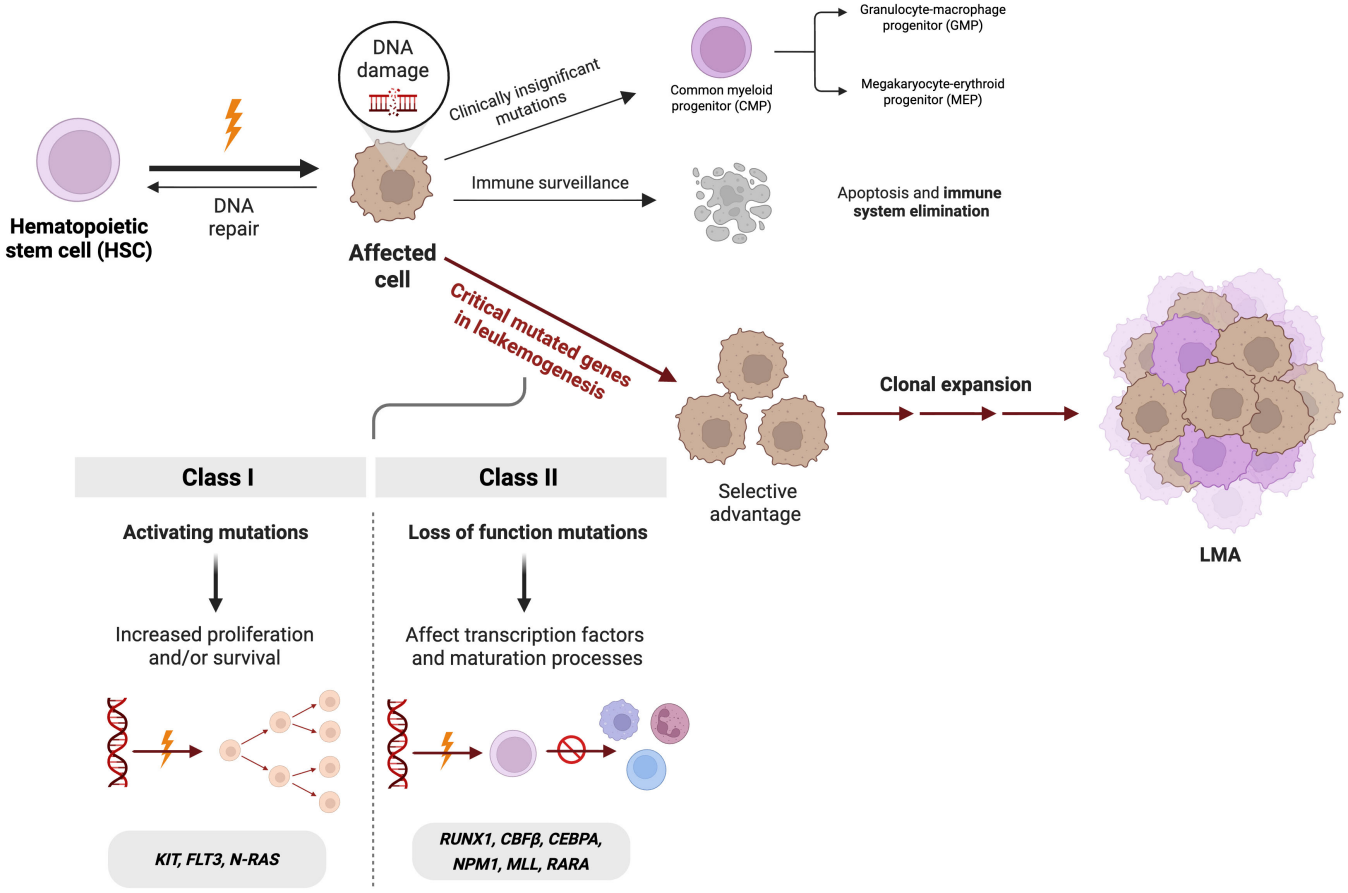
Key genetic alterations involved in leukemogenesis of acute myeloid leukemia (AML). Mutations in hematopoietic stem cells can disrupt some regulatory pathways, conferring a selective advantage that drives clonal expansion. In AML, Class I mutations (*KIT, FLT3, N-RAS*) increasing proliferation and survival of the tumor cells and Class II mutations (*RUNX1, CBFβ, CEBPA, NPM1, MLL, RARA*) impairing differentiation. *Figure created with*BioRender.com.

The development of AML is commonly explained by a two-class model of mutations. Class I mutations, also known as activating lesions, lead to dysregulated signaling cascades that enhance the proliferation and survival of hematopoietic progenitor cells (including *KIT*, *FLT3*, and *NRAS*). In contrast, Class II mutations impair hematopoietic differentiation through loss-of-function alterations in essential transcription factors or their cofactors, disrupting normal lineage commitment and maturation processes (including *RUNX1*, *CBFβ*, *CEBPA*, *NPM1*, *MLL*, and *RARA*) (**Figure 1**) (64, 65).

FLT3 mutations, particularly FLT3-ITD, lead to the constitutive activation of tyrosine kinase signaling pathways, driving uncontrolled proliferation and survival of leukemic cells. Mutations in RAS oncogenes, present in approximately 10% to 15% of AML cases, include activating alterations in NRAS, KRAS, PTPN11, and NF1, which lead to abnormal proliferative signaling through the MAPK and PI3K pathways. While the prognostic impact of RAS mutations remains debated, their acquisition or clonal expansion, much like FLT3 mutations, is frequently observed during the transition from myelodysplastic syndromes (MDS) to AML, typically correlating with a poor prognosis (64).

Alterations in NPM1 cause aberrant cytoplasmic localization of nucleophosmin, which disrupts ribosomal biogenesis and cellular differentiation. Normally, NPM1 shuttles between the nucleus and cytoplasm, playing a key role in chromatin remodeling, genomic stability, and ribosome biogenesis. However, its mutant form disrupts interaction with ARF, impairing the p53 pathway and thereby enabling the uncontrolled survival and proliferation of myeloid cells (66). Similarly, CEBPA encodes a transcription factor that functions as a tumor suppressor, inhibiting proliferation and driving neutrophil differentiation. CEBPA mutations, found in 7-11% of AML cases, occur as either single mutations (~45%) or double mutations (~55%) (66–68).

Mutations in *IDH1* and *IDH2* are gain-of-function alterations that produce the oncometabolite 2-hydroxyglutarate (2-HG). Elevated 2-HG levels inhibit α-ketoglutarate–dependent enzymes, including the TET family of DNA demethylases, leading to widespread DNA and histone hypermethylation (66). This epigenetic dysregulation blocks normal hematopoietic differentiation and contributes to the expansion of immature myeloid progenitors. Additionally, AML cells harboring IDH mutations often show increased dependence on anti-apoptotic pathways, such as BCL-2 (67). *TET2* is an epigenetic regulator that catalyzes the conversion of 5-methylcytosine to 5-hydroxymethylcytosine, facilitating DNA demethylation and normal gene expression during hematopoiesis. Loss-of-function mutations in *TET2*, which frequently occur independently of IDH mutations, disrupt DNA demethylation, resulting in aberrant self-renewal of hematopoietic stem cells, impaired differentiation, and genomic instability. This epigenetic alteration contributes to leukemogenesis and can cooperate with other mutations to drive AML development (66, 67).

Mutations in DNMT3A and ASXL1 further contribute to AML pathogenesis. DNMT3A, a DNA methyltransferase, plays an essential role in maintaining DNA methylation and regulating gene expression. Loss-of-function mutations in DNMT3A result in disrupted gene regulation and impaired differentiation, thereby promoting leukemogenesis (65). ASXL1, encoding a chromatin remodeling protein, is also frequently mutated in AML. These loss-of-function mutations affect chromatin regulation, altering gene expression and cellular differentiation. Both DNMT3A and ASXL1 mutations are associated with a poor prognosis, as they drive clonal expansion and disease progression (68).

Although less frequent, TP53 mutations have a significant impact on AML prognosis. Mutant TP53 leads to defective DNA damage response, increased genomic instability, and chemoresistance. In AML, these mutations often produce a truncated, defective p53 protein, impairing cell cycle arrest and apoptosis, allowing genetically unstable cells to proliferate uncontrollably (67).

Additionally, chromosomal rearrangements such as RUNX1-RUNX1T1, CBFB-MYH11, and PML-RARA play pivotal roles in AML subtypes. RUNX1-RUNX1T1, resulting from t (8, 21), interferes with RUNX1 function, blocking myeloid differentiation and altering gene expression. Similarly, CBFB-MYH11, formed by inv (16) or t (16, 16), disrupts the core-binding factor complex, impairing myeloid maturation and leading to the M4 AML subtype. PML-RARA, characteristic of acute promyelocytic leukemia (APL), disrupts the retinoic acid receptor (RAR) pathway, blocking transcriptional activation required for differentiation (66).

These genetic alterations often interact synergistically, creating a permissive environment for leukemogenesis through cumulative disruptions in cell signaling, epigenetic regulation, and differentiation.

## Integrating molecular data into the classification and management of AML

Building on the knowledge of leukemogenic mechanisms and recurrent somatic mutations, current classification systems aim to reflect this biological diversity by integrating genomic data into diagnostic and prognostic criteria. The World Health Organization (WHO, 2022) classification defines 11 distinct AML subgroups primarily based on specific genetic abnormalities. The European LeukemiaNet (ELN, 2022) builds upon this structure, identifying 14 molecularly defined groups with a stronger emphasis on risk stratification and clinical management. In parallel, the International Consensus Classification (ICC, 2022) delineates 18 recognized entities, further refining disease categorization (69–72).

Nowadays, molecular panels and cytogenetic analyses have become essential components of the diagnostic workup for myeloid neoplasms. Comprehensive panels that include recurrently mutated genes associated with AML and other myeloid malignancies, combined with traditional cytogenetic assessment, provide critical information for accurate diagnosis, risk stratification, and therapeutic decision-making (70, 71). These integrated approaches are now considered mandatory in routine clinical practice, as they enable the identification of actionable mutations, guide patient management, and form the basis for precision medicine strategies in hematologic malignancies.

The mandatory integration of molecular panels and cytogenetic analyses in myeloid neoplasms not only refines diagnosis but also guides therapy by identifying actionable mutations. *FLT3* mutations can be targeted with midostaurin or gilteritinib, while *IDH1/2* mutations respond to ivosidenib or enasidenib, and BCL-2 inhibition with venetoclax is effective in cases with epigenetic dysregulation. Ongoing trials are exploring therapies directed at *TP53*, *KIT*, and other epigenetic regulators. Importantly, these molecular insights could also inform the development of mutation-informed CAR-T cell strategies, enabling the design of personalized immunotherapies for AML (69, 73).

**Figure 2:**
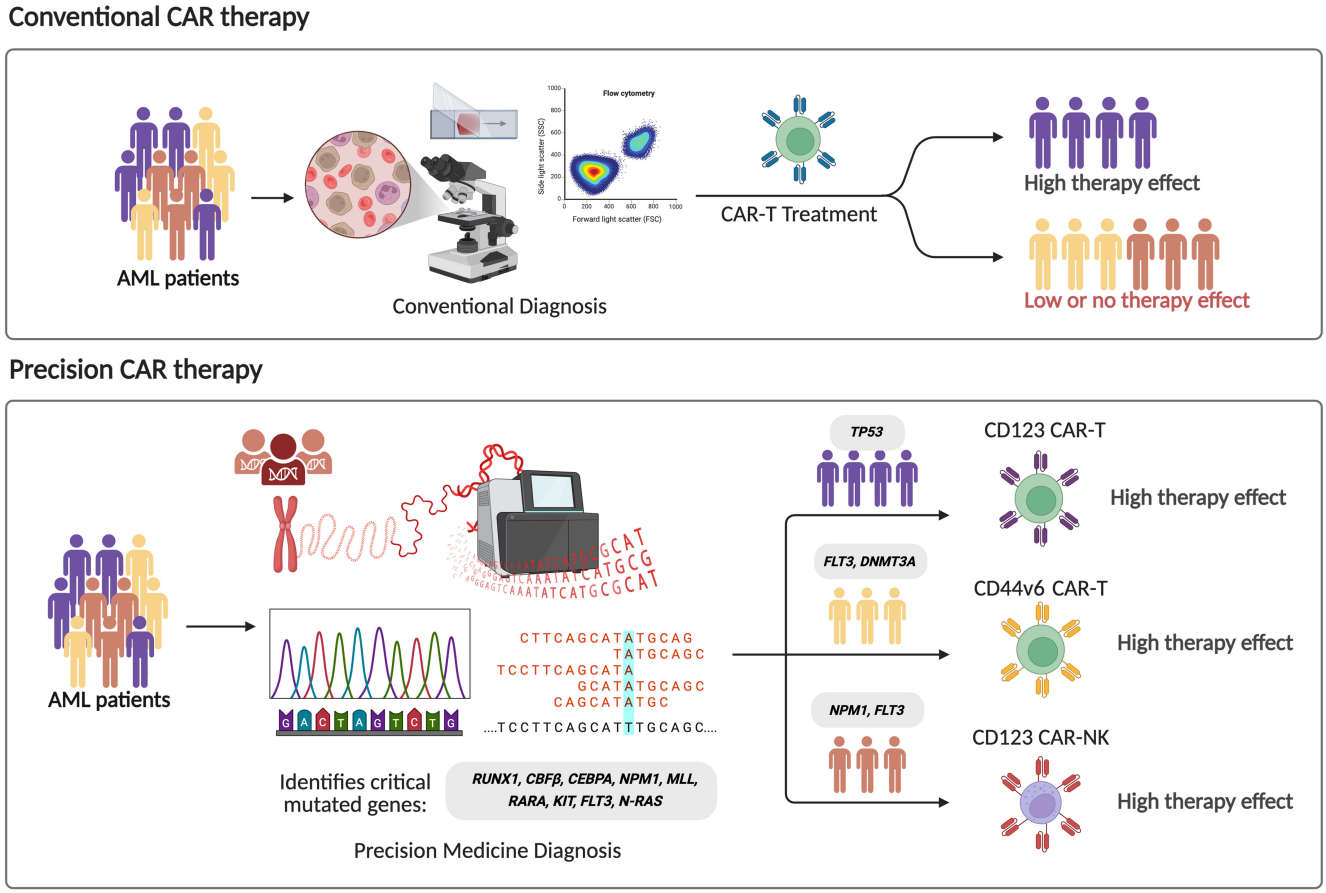
Comparison between conventional and precision CAR therapy strategies in acute myeloid leukemia (AML). In conventional strategy to CAR-based therapy, standard diagnostic methods such as morphology and flow cytometry are used, leading to heterogeneous therapeutic responses. In contrast, precision approaches integrate genomic profiling to identify critical driver mutations (e.g., *RUNX1*, *CBFB*, *CEBPA*, *NPM1*, *FLT3*, *DNMT3A*), enabling the target-matched CAR constructs, promoting higher treatment specificity and efficacy.

Currently, there are 75 registered clinical trials of CAR-T cell therapy for AML, of which 53 (71%) are Phase 1 or early Phase 1 trials, with CD33, CD123, CLL-1, FLT3 and NPM1c being the most frequently targeted antigens (69), as summarized in **Table 1**. Despite this growing number of studies, most of these trials do not incorporate the patients’ underlying molecular alterations into their design or analysis. This represents a critical limitation, as molecular heterogeneity is a defining feature of AML and significantly influences disease biology, progression, and therapeutic response. Distinct genetic alterations activate different signaling and epigenetic pathways, which could profoundly affect CAR-T cell efficacy, persistence, and resistance mechanisms. Therefore, integrating molecular profiling into CAR-T trial design is essential to move toward truly personalized and mutation-informed immunotherapies for AML (**Figure 2**).

**Table 1:** Comparative overview of major AML targets.

AML target	Expression	Safety	Clinical progress	Resistance	Reference
CD33	Expressed in more than 90% of leukemic blasts. Expressed in normal progenitor cells, myeloid cells, monocytes, tissue-resident macrophages	Myelosuppression, cytopenia and CRS	Phase I/II	Expressed in ex vivo expanded CAR-T cells. T cell exhaustion and fratricide	NCT07026942, NCT03473457 (74–77),
CD123	Overexpressed in FLT3-ITD and NPM1- mutated AMLs Expressed in normal hematopoietic cells	Severe CRS, on-target, off-tumor toxicity and capillary leak	Phase I	Toxicity limits dose and common relapse	NCT06420063, NCT06201247, NCT04678336 (78–80),
CLL-1	Expressed on 85%–92% of all AML classes. Expressed in differentiated myeloid cells. Not expressed in normal hematopoietic stem cells	Severe pancytopenia	Phase I	Cytopenias	NCT04219163, NCT06017258, NCT06128044 (81, 82),
FLT3	Overexpressed in AML FLT3 mutated (FLT3-TKD or FLT3-ITD). Lower levels in normal hematopoietic stem and progenitor cells	Minimal off-target toxicity	Phase I	Clonal evolution and drug resistance	NCT06786533, NCT06325748 (83),
NPM1c	A neoepitope result of mutation present in approximately 35% of AML patients. NPM1c-HLA-A2 complex presented on leukemic surface. Usually co-expression with CD123	Minimal off-tumor risk	No active trials	Low neoepitope density and HLA-A2 restriction	No active trials (84–86),

## Implications of genetic alterations in AML for CAR-T and CAR-NK therapies

AML is a highly complex disease, raising the question: How can CAR-T and CAR-NK therapies be leveraged to combat it? Genetic alterations in AML significantly influence the effectiveness of these immunotherapies by affecting antigen expression, immune evasion, and the tumor microenvironment. Mutations in key genes, such as FLT3, NPM1, TP53, TET2, DNMT3A, and CEBPA, as well as rearrangements involving KMT2A and RUNX1, can influence disease progression and response to treatment. Furthermore, clonal evolution and antigen loss present major hurdles, contributing to resistance and limiting the durability of CAR-based strategies. A deeper understanding of these genetic factors is crucial for refining immunotherapies and tailoring personalized approaches to improve AML outcomes.

**Table 2** summarizes genetic alterations reported in the literature on CAR cell therapies, highlighting their effects on treatment efficacy and resistance. Systematic evaluation of these mutations in the context of each patient’s unique genetic profile is critical for optimizing therapy. Understanding how specific alterations influence CAR cell responses can guide the development of safer and more effective strategies, improve target selection, and inform personalized treatment approaches for different patient subgroups.

**Table 2:** AML mutations and their effect on CAR therapies.

Therapy type	Antigen	Mutation	Mutation x CAR	Reference
CAR-T	FLT3	FLT3-ITD, FLT3-TKD	Targeted due to high mutation frequency; safety concerns due to expression in normal cells	(87–90)
CAR-NK	FLT3	FLT3-ITD, FLT3-TKD	Allogeneic FLT3-CAR-NK cells show efficacy with lower toxicity	(89, 91, 92)
CAR-T	CD44v6	FLT3, DNMT3A	Mutation increases CD44v6 expression; HMAs upregulate CD44v6, enhancing CAR-T efficacy	(87–90, 93–101)
CAR-T	CD123	NPM1, FLT3	High expression in leukemic stem cells; toxicity concern	(78, 79)
CAR-NK	CD123	NPM1, FLT3	Safer alternative to CAR-T with shorter lifespan	(102)
CAR-T	CD7	TP53 deletion, FLT3-ITD, SKAP2-RUNX1	Effective in high-risk AML; bridge to allo-HSCT	(103)
CAR-T	CD19	TET2	TET2 mutation enhances CAR-T cell efficacy and persistence but increases risk of hyperproliferation	(98–101 )
CAR-T	CD33, CD123, CD117	TP53	TP53-mutant AML cells exhibit resistance; impaired CAR-T proliferation and increased exhaustion; combinatorial strategies improve responses	(102, 104–112)
CAR-T	CD19	PIK3CD, PIK3R1	Robust responses in preclinical models of leukemia and melanoma	(113)
CAR-NK	NPM1 neoepitope	NPM1	Enhanced targeting and metabolic reprogramming; improved persistence	(84)
CAR-T	CD19	t(8;21) (RUNX1::RUNX1T1)	CD19 aberrantly expressed in ~78% of t(8;21) AML cases; CAR-T induces high remission rates but early relapse suggests need for consolidation with allo-HSCT	(114)
CAR-T	CD19	KMT2A rearrangements	Effective in inducing remission in CD19^+^ KMT2A-r leukemias; similar initial response rates but higher overall mortality; consolidation often required	(115)
CAR-T	CD19×FLT3 (bispecific)	KMT2A rearrangements	Bispecific CAR-Ts demonstrate strong *in vitro* and *in vivo* activity and may prevent antigen escape in KMT2A-r ALL	(116)
CAR-T	NKG2D	NRAS G12D, FLT3 N676K (KMT2A-r AML)	Activating mutations enhance NKG2D ligand expression, increasing susceptibility to NKG2D-CAR T killing; potentiated by panobinostat	(117)
CAR-T	CD19	RUNX1	Enhanced cytotoxicity; synergy with imatinib	(118)

## TP53

TP53 mutations are well established as drivers of tumor suppressor loss, treatment resistance, clonal evolution, and poor prognosis across hematologic malignancies. Their impact on cellular immunotherapy—including CAR-T and CAR-NK therapies—is increasingly recognized, but remains complex and context-dependent.

Patient-derived TP53 mutations can influence immune cell function and therapeutic efficacy. In AML, single-cell multi-omics revealed TP53 mutations in T and NK cells, with T cells showing elevated proliferative markers but reduced cytotoxicity and increased expression of inhibitory receptors such as PD-1, TIGIT, and TIM-3. Engineering CAR-T cells to carry clinically relevant TP53 mutations (e.g., Y220C, R175H) induced exhaustion, impaired cytokine secretion, and diminished tumor killing both *in vitro* and in PDX models. Restoration of mutant p53 to wild-type conformation rescued CAR-T functionality, reduced exhaustion, and improved survival, demonstrating that TP53-mutant T cells are a previously unrecognized driver of immune escape in AML (104).

Similarly, NK cells rely on p53 to maintain cytotoxic function and homeostasis. TP53 mutations in NK cells—often shared with malignant myeloid clones in AML or MDS—lead to increased DNA methylation, reduced expression of perforin, TNF-α, and killer immunoglobulin-like receptors (KIR), and impaired cytolytic activity. These alterations may compromise CAR-NK therapies, particularly autologous approaches, although epigenetic modulation (e.g., hypomethylating agents) can partially restore NK function (105). This underscores the importance of carefully screening NK cell donors in clinical trials to ensure optimal therapeutic efficacy and safety, even for off-the-shelf CAR-NK products. Donor-derived TP53 mutations could impair NK cell cytotoxicity, persistence, and immune surveillance, potentially reducing antitumor activity and compromising clinical outcomes. Systematic genotyping and functional assessment of donor NK cells can identify such defects, guide selection of optimal donors, and inform strategies for ex vivo modulation or epigenetic priming to restore NK cell fitness prior to infusion. Incorporating these measures is critical for standardizing CAR-NK products and maximizing their therapeutic potential, particularly in high-risk patient populations with hematologic malignancies.

Clinical experience also highlights the context-dependent impact of TP53 mutations. In lymphomas, patients harboring TP53 aberrations have achieved durable responses with multimodal strategies, including sequential CD19/CD22 CAR-T infusions, radiotherapy bridging, and maintenance therapy with chidamide, obinutuzumab, checkpoint inhibitors, and hypomethylating agents (106). Integration of autologous stem cell transplantation (ASCT) followed by CAR-T therapy has improved long-term outcomes and reduced severe toxicities in TP53-mutated cases (107, 108).

The influence of TP53 mutations on CAR-T therapy outcomes has also been explored in broader cohorts. In a phase 1/2 study of 115 patients with relapsed or refractory CD19-positive B-cell acute lymphoblastic leukemia (B-ALL), 93% achieved complete remission and 87% attained minimal residual disease negativity following CAR-T infusion (NCT03173417) (102). Among these, 75 patients underwent subsequent allogeneic stem cell transplantation (allo-HSCT), resulting in significantly improved leukemia-free survival (76.9% vs. 11.6%) and overall survival (79.1% vs. 32.0%) compared with CAR-T therapy alone. Multivariate analysis identified TP53 mutation as an independent predictor of inferior outcomes (hazard ratio, 0.235; 95% CI, 0.089–0.619) (102). These findings highlight that while CAR-T therapy achieves high initial response rates, TP53 alterations remain a major determinant of relapse risk and survival.

TP53 mutations can also drive clonal hematopoietic evolution post–CAR-T therapy, as illustrated by cases of therapy-related myelodysplasia following CAR-T–induced selective pressures (109). At the mechanistic level, TP53 deficiency has been shown to impair CAR-T cell efficacy in AML models. TP53-deficient AML cells exhibit prolonged interactions with CAR-T cells, leading to CAR-T exhaustion and inadequate tumor clearance. Transcriptomic analyses revealed upregulation of the mevalonate pathway in TP53-deficient AML cells and concurrent downregulation of the Wnt pathway in CAR-T cells (110). Pharmacologic inhibition of the mevalonate pathway or activation of Wnt signaling restored CAR-T cytotoxicity, identifying potential therapeutic strategies to overcome TP53-mediated immune resistance (110). Consistent with these findings, TP53-mutant AML and myelodysplastic neoplasms (MDS) show reduced CAR-T proliferation and increased exhaustion compared to wild-type counterparts (111). Promisingly, pre-treatment with hypomethylating agents such as decitabine has demonstrated potential to enhance CAR-T responses in patients with relapsed or refractory acute leukemia and TP53 mutations. In one report, decitabine priming significantly improved CAR-T cell efficacy, resulting in complete molecular remission in six patients (112).

Collectively, these findings underscore a dual role of TP53 mutations in cellular immunotherapy. Patient-derived TP53 alterations impair the fitness, cytotoxicity, and persistence of both T and NK cells, limiting the efficacy of autologous CAR-T and CAR-NK therapies. Tumor-intrinsic TP53 loss contributes to immune evasion and resistance, while clonal hematopoietic evolution may further challenge long-term outcomes. Conversely, targeted modulation of TP53—via reactivation or pathway correction—can restore immune-cell function, reduce exhaustion, and enhance antitumor potency. Integrating CAR-T/CAR-NK engineering, transplantation, metabolic or epigenetic modulators, and rational pathway targeting provides a comprehensive framework to overcome TP53-driven resistance, improving efficacy and durability in high-risk hematologic malignancies (119).

## DNMT3A

Mutations in DNMT3A within the patient’s hematopoietic compartment can impact CAR-T therapy by promoting expansion of progenitor and myeloid cells that chronically stimulate T cells, ultimately leading to functional impairment and reduced activation signatures. While Dnmt3a-deficient murine cells show enhanced antigen-specific T cell stimulation, chronic antigen exposure in patients likely drives T cell exhaustion, potentially compromising autologous CAR-T therapies derived from DNMT3A-mutant T cells (120).

In AML, DNMT3A mutations also influence therapeutic targeting. CD44v6 has emerged as a promising CAR-T cell target, particularly in cases with FLT3 or DNMT3A mutations, where its expression is markedly elevated. CAR-T cells directed against CD44v6 selectively lyse CD44v6-positive AML cells while sparing CD44v6-negative leukemic and normal hematopoietic cells. Treatment with hypomethylating agents, such as decitabine or azacitidine, further upregulates CD44v6 on DNMT3A-mutant AML cells, enhancing CAR-T recognition and cytotoxicity. This combination represents a promising strategy to improve outcomes in AML patients harboring DNMT3A or FLT3 mutations (93–95).

DNMT3A also directly regulates CAR-T cell biology. Genetic disruption of DNMT3A prevents methylation of genes critical for T cell differentiation, preserving a stem-like phenotype resistant to exhaustion. These modified CAR-T cells maintain proliferative capacity and effector function under prolonged antigen stimulation, resulting in superior tumor control in preclinical models. DNMT3A-mediated methylation therefore represents a key determinant of T cell fate, with implications for enhancing CAR-T persistence and antitumor efficacy by preserving naïve and memory-associated gene programs (96).

Collectively, DNMT3A plays a dual role in AML CAR-T therapy: patient-derived DNMT3A mutations can indirectly reduce CAR-T efficacy through chronic T cell stimulation and exhaustion, whereas manipulation of DNMT3A within CAR-T cells themselves can preserve stem-like, functional phenotypes and improve therapeutic outcomes. Moreover, DNMT3A mutations create actionable vulnerabilities, such as CD44v6 upregulation, which can be exploited with hypomethylating agents to optimize CAR-T cell targeting. These insights provide a rationale for integrating genetic, epigenetic, and combinatorial strategies to enhance CAR-T efficacy in DNMT3A-mutant AML.

## TET2

TET2 encodes a dioxygenase critical for DNA demethylation and broader epigenetic regulation, playing a central role in hematopoiesis. Mutations in TET2 occur in 7%–28% of adult AML patients and are associated with altered epigenetic landscapes that can affect immune function and therapeutic responses (97).

Patient-derived TET2 mutations can profoundly influence CAR-T therapy by affecting T cell fitness, differentiation, and immune surveillance. In a landmark case of chronic lymphocytic leukemia (CLL), lentiviral integration of the CAR transgene disrupted the TET2 gene in one T cell, creating a hypomorphic loss-of-function state (98). This TET2-disrupted T cell underwent massive clonal expansion, eventually accounting for 94% of the CD8^+^ CAR-T cell population at the peak of the response. These cells predominantly exhibited a central memory phenotype, characterized by high proliferative potential and long-term persistence, which is strongly associated with durable antitumor activity. The patient achieved complete remission, highlighting that TET2 disruption can enhance CAR-T efficacy by promoting memory formation, limiting terminal differentiation, and sustaining effector function (98). Experimental knockdown of TET2 in CAR-T cells recapitulated this effect, confirming that TET2 modulation directly contributes to enhanced proliferation, persistence, and functional potency of CAR-T cells (99).

In AML, TET2 loss may further influence therapy by altering the antigenic landscape of malignant cells. Epigenetic dysregulation driven by TET2 mutations generates a distinct repertoire of peptides presented on major histocompatibility complex (MHC) molecules, increasing the immunogenicity of AML cells. These TET2-associated neoantigens create opportunities for CAR-T cells to more effectively recognize and eliminate malignant and pre-malignant cells. Leveraging this increased antigenicity could improve tumor clearance, reduce relapse risk, and inform the design of combinatorial immunotherapies or CAR-T engineering strategies tailored to TET2-mutant AML (100).

Similar to CAR-T cells, TET2 mutations in the patient’s hematopoietic compartment can also impact the function and fitness of natural killer (NK) cells, with implications for CAR-NK therapy. In myelodysplastic syndromes (MDS), TET2-mutated clones frequently coexist with NK cells harboring the same mutation. These NK cells exhibit phenotypic defects, including increased global DNA methylation and reduced expression of cytolytic effectors such as perforin, TNF-α, and killer immunoglobulin-like receptors (KIR), resulting in impaired antitumor activity. *In vitro*, inhibition of TET2 in NK cells from healthy donors reproduces these functional defects, while treatment with hypomethylating agents such as azacitidine partially restores NK cell cytotoxicity and IFN-γ production, highlighting the reversible nature of these epigenetic impairments (101).

Collectively, these observations highlight the dual relevance of TET2 in cellular immunotherapy. Patient-derived TET2 mutations can impair endogenous T and NK cell fitness, reducing immune surveillance and potentially limiting the efficacy of autologous CAR-T and CAR-NK therapies. Conversely, targeted modulation of TET2 within engineered CAR-T cells can enhance memory differentiation, limit exhaustion, and boost antitumor potency. In NK cells, pharmacologic interventions such as hypomethylating agents may restore cytotoxic function, further supporting therapeutic efficacy. Additionally, TET2-associated neoantigens in AML represent actionable targets for immunotherapy. Together, these insights provide a framework for integrating genetic, epigenetic, and combinatorial strategies to optimize the performance and durability of CAR-based therapies in TET2-mutant hematologic malignancies.

## FLT3

FMS-like tyrosine kinase 3 (FLT3) is a transmembrane receptor tyrosine kinase highly expressed in acute myeloid leukemia (AML), particularly in cases harboring internal tandem duplication (FLT3-ITD) or tyrosine kinase domain (FLT3-TKD) mutations, which occur in approximately 30% of patients. These mutations drive constitutive signaling that promotes leukemic proliferation and survival, correlating with poor prognosis (77). Unlike CD19 in acute lymphoblastic leukemia (ALL), FLT3 is also expressed at lower levels in normal hematopoietic stem and progenitor cells (HSPCs), raising concerns about on-target, off-tumor toxicity for CAR-based therapies. Nonetheless, the strong association between FLT3 alterations and adverse clinical outcomes makes it a compelling therapeutic target.

FLT3 inhibitors have demonstrated clinical efficacy, yet relapse due to clonal evolution and drug resistance remains common. In this context, FLT3-directed CAR-T and CAR-NK cell therapies have emerged as promising immunotherapeutic strategies. Preclinical studies demonstrated that both CD8^+^ and CD4^+^ T cells engineered to express FLT3-specific CARs exhibit potent cytotoxicity against AML cell lines and primary blasts expressing either wild-type or mutant FLT3 (87). Notably, treatment with FLT3 inhibitors such as crenolanib increases FLT3 surface expression on AML cells, thereby enhancing recognition and killing by FLT3-CAR T cells *in vitro* and *in vivo*. However, these same CAR-T cells also recognize normal HSCs, disrupting hematopoiesis in preclinical assays—an observation suggesting that adoptive FLT3-CAR T therapy may need to be followed by CAR-T cell depletion and subsequent allogeneic HSC transplantation for marrow reconstitution (87).

Comparative analyses between FLT3-directed CAR-T and bispecific T-cell engagers (BiTE^®^) have shown both platforms to mediate potent cytotoxicity against FLT3^+^ AML targets. However, *in vivo*, FLT3-CAR T cells achieved superior persistence, proliferation, and overall survival benefit in murine models, likely due to the integrated co-stimulatory domain within the CAR construct (88).

Beyond CAR-T, preclinical studies have explored FLT3-targeted CAR-NK approaches. One study developed off-the-shelf, allogeneic NK cells engineered with a FLT3-specific CAR and secreting soluble interleukin-15 (IL-15), demonstrating enhanced cytotoxicity and interferon-gamma secretion against FLT3^+^ AML cell lines compared to NK cells lacking the CAR or IL-15 secretion (89). Another study reported the preclinical development of a UniCAR-T platform targeting FLT3, which efficiently eliminated AML cell lines and primary AML samples *in vitro* and showed *in vivo* efficacy in murine xenotransplant models (90). These findings underscore the translational potential of both CAR-T and CAR-NK strategies against FLT3-positive AML.

In clinical translation, early-phase trials are assessing the safety of anti-FLT3 CAR-T therapy in relapsed or refractory AML, including both adult and pediatric populations. The study completion is estimated to be 2027 (NCT06786533). Emerging case reports also underscore the complex interplay between prior intensive therapies, clonal hematopoiesis, and the emergence of secondary AML harboring FLT3-ITD mutations following CAR-T exposure, emphasizing the need for long-term hematopoietic monitoring (121).

FLT3 has also been successfully targeted using CAR-engineered NK cells. NK-92 cells expressing a FLT3-specific CAR with a CD28–CD3ζ signaling domain demonstrated strong cytolytic activity against FLT3^+^ B-ALL and AML cells *in vitro* and robust antileukemic efficacy in xenograft models. Incorporation of an inducible caspase-9 (iCasp9) suicide switch provided an added safety layer to mitigate potential off-target hematopoietic toxicity (91). More recently, SENTI-202, a logic-gated CAR-NK product designed to target FLT3 and/or CD33 while sparing EMCN^+^ healthy HSPCs, has shown encouraging preliminary data in a Phase I trial (NCT06325748). In treated relapsed/refractory AML patients, SENTI-202 induced deep molecular remissions and significant reductions in leukemic stem cells (LSCs), while preserving normal hematopoiesis and enabling immune reconstitution (92).

Collectively, these data establish FLT3 as a highly relevant but challenging target for cellular immunotherapy. FLT3-CAR T and CAR-NK platforms have demonstrated potent preclinical efficacy and early clinical feasibility, with rational combinatorial strategies—such as tyrosine kinase inhibition, immune checkpoint blockade, and logic-gated receptor design—enhancing both selectivity and persistence. Optimizing safety mechanisms to mitigate HSPC toxicity and integrating donor or off-the-shelf NK cell platforms will be critical for translating FLT3-targeted CAR therapies into durable and safe treatment options for high-risk AML.

## NPM1

*NPM1* is one of the most frequently mutated genes in AML, with a four-nucleotide duplication occurring in approximately 30–35% of adult patients. The cytoplasmic localization of NPM1c allows presentation of neoepitopes via HLA, particularly HLA-A*0201, making the NPM1c-HLA complex a potential target for CAR-T or memory NK cell therapies (86). A limitation of this approach is the relatively low density of NPM1 neoepitopes available for recognition by anti-NPM1c/HLA-A2 CAR-T cells, which may necessitate additional strategies to achieve robust T-cell activation. One potential solution is the development of dual CAR-T cells co-expressing an anti-NPM1c/HLA-A2 CAR along with an anti-CD123 co-stimulatory receptor (CCR) lacking a signaling domain, designed to enhance T-cell activation without inducing off-target signaling (122).

Despite the generally favorable outcomes reported in large cohorts of AML patients harboring *NPM1* mutations, disease relapse and progression ultimately lead to mortality in roughly half of these individuals (123, 124). Interestingly, a study previously reported that CD123 is present on leukemic stem cells putatively harboring *NPM1* mutations (125). This same group, years later, has demonstrated that CD123 is prominently displayed on *NPM1*-mutated AML cells at both initial diagnosis and relapse, with particularly elevated expression in cases harboring concurrent *FLT3-ITD* mutations. The group concluded that putative *NPM1*-mutated CD34^+^CD38^-^ leukemic stem cells consistently express high levels of CD123, a pattern further amplified in cases with concurrent *FLT3* mutations, highlighting *NPM1*-mutated AML as a particularly promising target for anti-CD123 immunotherapies (85). CD123-targeting CAR-T cells have demonstrated potent anti-leukemic activity; however, they also pose risks of on-target, off-tumor toxicity, as CD123 is expressed in normal hematopoietic cells. Clinical trials, such as NCT04318678, have reported promising responses but also significant myelosuppression, underscoring the need for strategies to improve safety (46). In contrast, CD123-CAR-NK cells present a potentially safer alternative due to their shorter lifespan and lower toxicity risks (126).

The development of memory-like NK cells with a neoepitope-specific chimeric antigen receptor (CAR) has emerged as a promising strategy for targeting AML with NPM1 mutations. Recent paradigm-shifting studies have demonstrated that NK cells can acquire innate immunological memory following brief stimulation with IL-12 and IL-18, resulting in the generation of cytokine-induced memory-like (CIML) NK cells. hese CIML NK cells display enhanced cytotoxicity and persistence, showing encouraging efficacy in early-phase clinical trials for relapsed or refractory AML (127, 128). In other study, the authors engineered CIML NK cells with CAR targeting the NPM1 mutation in AML. The CAR-CIML NK cells demonstrated potent and selective cytotoxicity against NPM1-mutated AML cells while sparing healthy hematopoietic cells, thereby minimizing off-target toxicity. Single-cell RNA sequencing and mass cytometry analyses revealed that CAR-transduced CIML NK cells upregulate genes involved in cell proliferation, protein folding, immune activation, and key metabolic pathways, promoting tumor-specific, CAR-dependent recognition and cytotoxicity (84). These findings position CAR-NK therapy as a targeted and potentially safer alternative for NPM1-mutated AML.

## Gene fusions

In addition to recurrent somatic mutations, gene fusions represent a critical class of genetic alterations in AML, playing a central role in leukemogenesis by disrupting normal transcriptional programs, driving aberrant proliferation, and impairing differentiation. These fusions, such as *RUNX1-RUNX1T1* and *KMT2A* rearrangements, are key diagnostic markers and are incorporated into contemporary AML classification systems, including WHO, ELN, and ICC, for risk stratification and treatment guidance.

Around 78% of AML patients with *t* (8,21) exhibit CD19 expression, highlighting CD19 as a promising therapeutic target for CAR-T cell–based strategies. In this context, a prospective phase II clinical trial (NCT03896854) investigated the safety and therapeutic efficacy of CD19-directed CAR-T cells in a cohort of 10 patients with relapsed CD19-positive *t* (8,21) AML. All ten patients treated with CAR-T cells achieved complete remission, corresponding to a 100% response rate, and 60% of them reached molecular MRD-negative remission. Following CAR-T therapy, the *RUNX1::RUNX1T1* fusion transcript levels demonstrated a median decrease too (114). In another study, 3 R/R t(8;21) AML patients with aberrant CD19 expression also received autologous CAR-T cell infusion. All patients achieved CD19 negativity within two weeks after CD19 CAR-T cell infusion, confirming the therapy’s short-term efficacy in relapsed/refractory *t*(8;21) AML with aberrant CD19 expression. However, early relapse occurred in some cases, suggesting that allogeneic hematopoietic stem cell transplantation (allo-HSCT) should be promptly performed following CAR-T therapy to sustain remission and minimize relapse risk (129).

Rearrangements involving the KMT2A gene, formerly known as *mixed-lineage leukemia (MLL)*, represent some of the most frequent chromosomal alterations found in both AML and ALL, encompassing a wide range of fusion partners. Leukemias harboring KMT2A rearrangements typically exhibit an aggressive clinical course, marked by rapid disease onset and progression, and are associated with significantly poorer outcomes compared to non–KMT2A-rearranged cases (130). Immunotherapeutic strategies, such as CAR-T cell therapy and antibody-drug conjugates, are being explored to target specific cell surface markers present in *KMT2A* rearrangements leukemia cells.

A recent study evaluated the impact of high-risk cytogenetic abnormalities on the outcomes of children and young adults with CD19-positive B-cell precursor acute lymphoblastic leukemia (B-ALL) or lymphoblastic lymphoma treated with CD19-directed chimeric antigen receptor (CAR) T-cell therapy (NCT01626495, NCT02435849, NCT02374333, NCT02228096, and NCT02906371) (115). The analysis included patients with a broad range of cytogenetic profiles, classified as high-risk (e.g., *KMT2A* rearrangements, Ph+, Ph-like, hypodiploidy), standard-risk, or favorable subtypes (e.g., hyperdiploidy, *ETV6-RUNX1*). Overall, 94% of patients achieved complete remission following CAR-T cell infusion, and no statistically significant differences in initial response rates were observed across cytogenetic subgroups. Patients with *KMT2A* rearrangements achieved similar remission rates but showed higher overall mortality, underscoring their aggressive disease course. Thus, while CD19 CAR-T therapy can induce durable responses in high-risk cases, consolidation with allogeneic stem cell transplantation may be necessary to sustain long-term remission (115).

Additionally, other study demonstrated that mutations in FLT3 and NRAS have been shown to reshape chromatin accessibility and transcriptional programs in KMT2A-rearranged AML, directly influencing immune sensitivity. Notably, *NRAS G12D* and *FLT3 N676K* mutations induced immune-related transcriptional programs, leading to increased expression of NKG2D ligands on leukemia cells, making them more susceptible to NKG2D-CAR T cell-mediated killing. Furthermore, treatment with the histone deacetylase inhibitor LBH589 (panobinostat) upregulated NKG2D ligands, enhancing the efficacy of CAR T cell therapy, especially in cells expressing *NRAS G12D* (117). These findings suggest that activating mutations in *FLT3* and *NRAS* not only drive leukemogenesis but also create vulnerabilities that can be targeted by immunotherapies, offering potential therapeutic strategies for *KMT2A*-rearranged acute leukemia. Another study explored an alternative therapeutic approach for KMT2A-rearranged acute lymphoblastic leukemia (ALL) by evaluating bispecific CD19xFLT3 CAR-T cells (116). They observed strong antitumor activity of bispecific CD19xFLT3 CAR-T cells both *in vitro* and *in vivo* against KMT2A-rearranged ALL, and propose that this dual-targeting strategy could help mitigate antigen escape in these high-risk leukemias (116).

## Other genetic alterations

A recent study explored the functional impact of single-nucleotide variants (SNVs) in key T cell signaling genes using base-editing screens in primary human T cells. High-throughput base-editing screens have identified gain-of-function (GOF) mutations in genes such as PIK3CD, PIK3R1, and LCK, which enhance T-cell signaling, cytokine production, and tumor cell lysis (113). Introduction of GOF mutations in *PIK3CD* and *PIK3R1* into T cells expressing either tumor-specific T cell receptors or various generations of CD19-directed CAR-T cells enhanced antigen-specific signaling, cytokine production, and tumor cell killing. Notably, CAR-T cells engineered with *PIK3CD* GOF variants exhibited superior effector function and leukemia cell cytotoxicity compared to standard CAR-T cells, highlighting the potential of precise genomic engineering of T cells to optimize immunotherapeutic efficacy (113). Through integrated multi-omic analyses, the CBFA2T3-GLIS2 (C/G) fusion was shown to drive transformation of cord blood hematopoietic stem and progenitor cells (HSPCs) into aggressive acute megakaryoblastic leukemia, recapitulating the transcriptomic, morphological, and immunophenotypic features of the disease (131). These analyses also identified fusion-specific targets, including folate receptor α (FOLR1), which could be leveraged for CAR-T cell-based therapeutic strategies, although potential off-tumor toxicity remains a concern.

Since CD7 is expressed in approximately 30% of AML cases while being absent on normal myeloid and erythroid cells, it represents a promising target for immunotherapy. A groundbreaking clinical case demonstrated the first successful use of CD7 CAR-T cell therapy in a patient with relapsed/refractory acute myeloid leukemia (r/r AML) carrying high-risk mutations, including TP53 deletion, FLT3-ITD, and SKAP2-RUNX1 fusion (NCT04762485). Following treatment, the patient exhibited complete blast clearance, karyotype normalization, and a significant reduction in mutation burden. Notably, CD7 CAR-T therapy effectively reduced the leukemic burden. It served as a bridge to allo-HSCT with manageable toxicity, underscoring its therapeutic potential for CD7-positive r/r AML (103).

Finally, promising evidence supports the use of CD19 CAR-T cell therapy in RUNX1-mutated blast-phase chronic myeloid leukemia (BP-CML), a high-risk condition with poor prognosis. Ex vivo studies showed that CD19 CAR-T cells efficiently and specifically eliminated RUNX1-mutated BP-CML blasts, affecting both lymphoid and myeloid compartments. Notably, these CAR-T cells outperformed imatinib, including in cases carrying the ABL1-T315I resistance mutation. Furthermore, the combination of CD19 CAR-T therapy with imatinib enhanced leukemia cell eradication, suggesting a synergistic benefit. Overall, these findings highlight CD19 CAR-T cells as a promising therapeutic strategy for BP-CML patients with RUNX1 mutations, especially those resistant to standard treatments (118).

**Figure 3:**
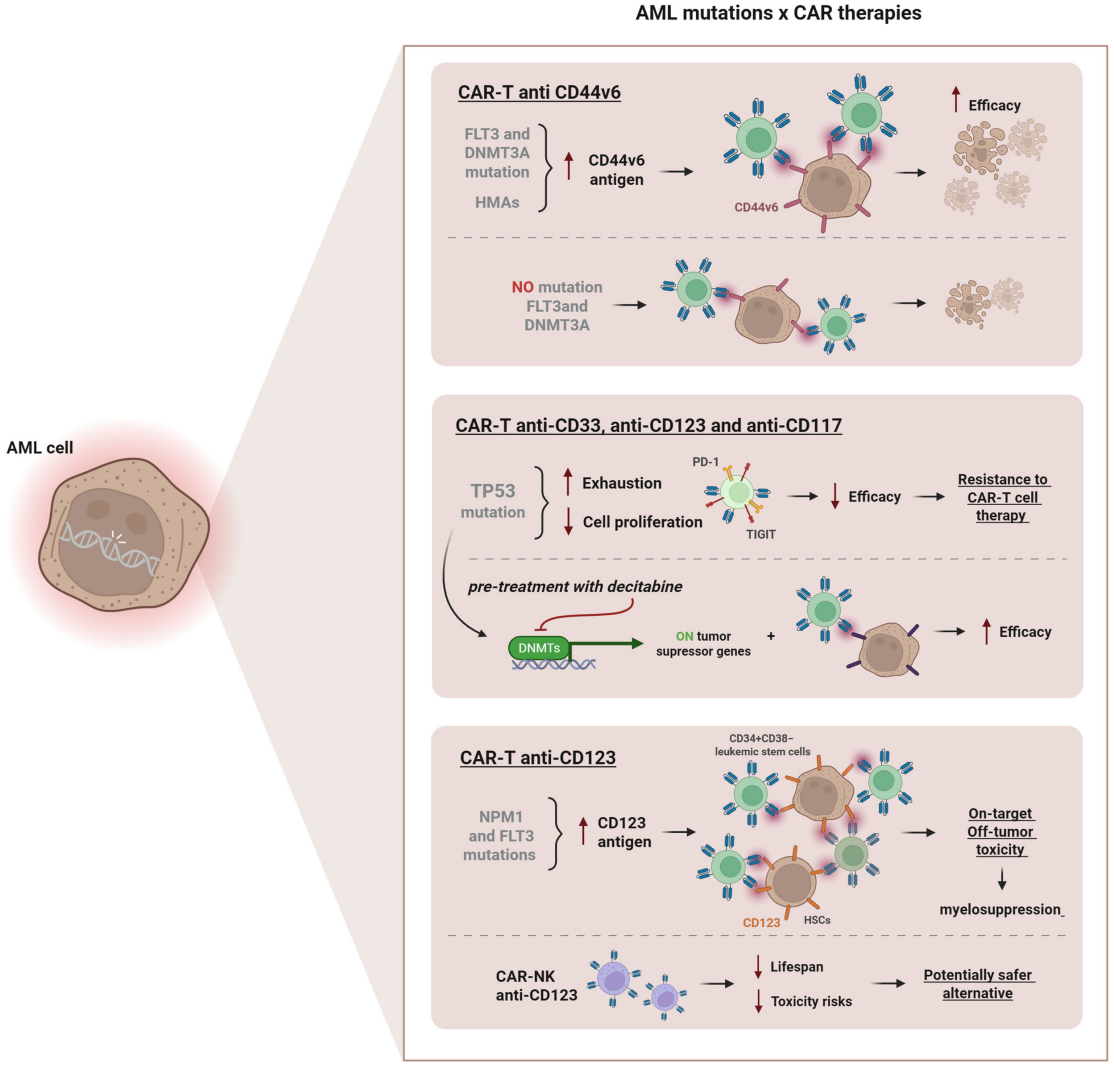
Influence of AML-associated mutations on CAR therapies. Mutations in *FLT3* and *DNMT3A* increase CD44v6 antigen expression, enhancing CAR-T efficacy. Pre-treatment with hypomethylating agents (HMAs), such as decitabine or azacitidine further upregulates CD44v6 levels, improving anti-leukemic activity. *TP53* mutations promote T cell exhaustion and upregulate inhibitory receptors (PD-1, TIGIT), reducing therapeutic efficacy. Pre-treatment with decitabine can restore tumor suppressor gene activity, improving CAR-T anti-CD33, anti-CD123 and anti-CD117 function. *NPM1* and *FLT3* mutations increase CD123 expression, but targeting the CD123 CAR-T can cause on-target/off-tumor toxicity and myelosuppression due to antigen expression on hematopoietic stem cells. CAR-NK anti-CD123 cells represent a safer alternative, reducing lifespan and toxicity risks.

Some of the key molecular targets, mechanisms of resistance and therapeutic strategies discussed above are illustrated in **Figure 3**.

## Translational barriers and practical considerations

While the concept of mutation-informed CAR-based therapy in AML holds strong scientific and clinical appeal, its translation into routine clinical practice remains constrained by several practical limitations. The integration of multi-omic profiling—including genomic, transcriptomic, and epigenetic data—into patient care is not yet feasible at scale. AML’s rapid progression often precludes the time required for comprehensive molecular characterization and subsequent customized CAR manufacturing. Furthermore, high sequencing costs, limited access to advanced diagnostic platforms, and variability in data interpretation represent additional barriers, particularly in resource-limited settings.

Manufacturing and logistical challenges are equally significant. The development of personalized CAR-T or CAR-NK therapies demands rapid cell procurement, engineering, and quality-controlled release, all within tight clinical timelines. While innovations such as off-the-shelf allogeneic CAR platforms, logic-gated designs, and universal donor NK cells may mitigate some of these constraints, they introduce new safety, regulatory, and scalability considerations. Ensuring batch consistency, preventing graft-versus-host effects, and maintaining persistence and potency remain central challenges.

Effective patient selection represents another key hurdle. Linking mutational profiles to actionable antigen expression requires integrated diagnostic pipelines that combine molecular profiling, flow cytometry, and functional immune assays. Realistically, precision integration will depend on the establishment of streamlined testing frameworks that can identify eligible patients within clinically relevant timeframes.

Finally, **r**egulatory and ethical frameworks must evolve to accommodate this new therapeutic paradigm. Personalized, mutation-informed cellular products challenge conventional approval models and demand adaptive oversight for manufacturing, safety monitoring, and long-term follow-up. Harmonized regulatory pathways and collaborative data-sharing initiatives will be essential to accelerate responsible clinical translation.

Together, these barriers emphasize that while mutation-informed CAR therapy represents an exciting frontier, practical implementation requires parallel innovation in diagnostics, manufacturing, and regulation. Bridging these gaps will be critical to transform precision immunotherapy from concept to standard-of-care.

## Conclusion and perspectives

From a clinical perspective, car-based therapies are expanding beyond traditional targets, demonstrating activity in challenging contexts. these findings reinforce the potential of combinatorial strategies—integrating car therapy with kinase inhibitors (e.g., imatinib), immune checkpoint blockade, or allogeneic hsct—to overcome resistance and improve outcomes.

looking forward, several key priorities should guide the field:

1. Enhancing persistence and preventing exhaustion through metabolic and epigenetic modulation, such as DNMT3A-modified CAR-Ts or reprogrammed ML-NK cells.
2. Improving precision and safety by developing neoepitope-specific CAR constructs (e.g., NPM1c-HLA-A2 CAR-T) that spare normal hematopoiesis.
3. Expanding combinatorial and multimodal approaches that leverage small-molecule inhibitors, checkpoint blockade, or cytokine support to augment CAR efficacy.
4. Optimizing translation and accessibility, emphasizing scalable, allogeneic, and cost-effective CAR platforms supported by rapid molecular diagnostics.

Collectively, these efforts define a realistic roadmap for the next generation of precision CAR-T and CAR-NK therapies in AML. Although the field remains in its early stages and many translational questions persist, growing evidence supports the feasibility and transformative potential of mutation-guided cellular immunotherapy. Continued integration of genomic insight, rational engineering, and clinical innovation will be key to achieving durable, safe, and accessible treatments for high-risk hematologic malignancies.
